# Successful application of technetium-99m-labeled octreotide acetate scintigraphy in the detection of ectopic adrenocorticotropin-producing bronchial carcinoid lung tumor: a case report

**DOI:** 10.1186/1752-1947-4-323

**Published:** 2010-10-18

**Authors:** Armaghan Fard Esfahani, Maryam Chavoshi, Mohammad Hadi Noorani, Mohsen Saghari, Mohammad Eftekhari, Davood Beiki, Babak Fallahi, Majid Assadi

**Affiliations:** 1 Research Institute for Nuclear Medicine, Shariati Hospital, Tehran University of Medical Sciences, Tehran, Iran; 2 Bushehr Research Center for Nuclear Medicine, The Persian Gulf Biomedical Sciences Institute, Bushehr University of Medical Sciences, Bushehr, Iran

## Abstract

**Introduction:**

The diagnostic efficacy of somatostatin receptor scintigraphy labeling with 111 indium in the localization of tumors has been assessed in a limited number of patients with contradictory outcomes. Here, we describe the case of a patient with an ectopic adrenocorticotropic hormone-producing bronchial carcinoid tumor diagnosed preoperatively using technetium-99m-labeled octreotide acetate scintigraphy.

**Case presentation:**

A 29-year-old Asian man presented to our hospital with the typical clinical features of Cushing's syndrome, which he had had for a duration of 18 months. The results of a biochemical evaluation revealed he had adrenocorticotropic hormone-dependent Cushing's syndrome. The results of a spiral abdominal computed tomography scan showed he had bilateral adrenal hypertrophy. A magnetic resonance image of the patient's brain showed he had a normal hypophysis. Whole body technetium-99m-labeled octreotide acetate scintigraphy was performed to check for the presence of an ectopic adrenocorticotropic hormone-producing tumor. The scan results showed a small focal increase in uptake in the lower lobe of our patient's right lung, just above his diaphragm. A spiral chest computed tomography scan also revealed a small non-specific lesion in the same region. A transthoracic biopsy was then performed. Pathological evaluation confirmed the diagnosis of a carcinoid tumor, of the adrenocorticotropic hormone-producing type. After surgical removal, the patient's symptoms resolved and significant clinical improvement was achieved.

**Conclusions:**

This case report shows that technetium-99m-labeled octreotide acetate scintigraphy can effectively detect an ectopic adrenocorticotropic hormone-producing bronchial carcinoid.

## Introduction

The ectopic secretion of adrenocorticotropic hormone (ACTH) from nonpituitary tumors causes approximately 10% cases of Cushing's syndrome [1]. In some patients, where the presence of an ectopic tumor has been considered as the cause of Cushing's syndrome, localization of the tumor has been difficult using modalities such as computed tomography (CT) and magnetic resonance imaging (MRI) of the patient's chest and abdomen, leaving palliative chemical or surgical adrenalectomy as the available treatment options [2].

Somatostatin receptor scintigraphy (SRS) using 111 indium (In)-pentetreotide and 18F-fluorodeoxyglucose positron emission tomography (FDG-PET) are the functional techniques currently used to detect ectopic ACTH-secreting lesions. However, the diagnostic efficacy of SRS labeling with 111In in the localization of such tumors has only been assessed in a limited number of patients, with contradictory outcomes [3].

We describe a case of a patient with an ectopic ACTH-producing bronchial carcinoid tumor diagnosed preoperatively using technetium-99m-labeled octreotide acetate scintigraphy.

## Case presentation

A 29-year-old Asian man presented to our hospital with upper and lower extremity weakness, significant weight gain (20 kg over 18 months), dyspnea, insomnia, early-morning awakening, psychiatric symptoms (illusions, impaired concentration and memory, inappropriate laughter and crying attacks), and erectile dysfunction. Physical examination revealed the typical clinical features of Cushing's syndrome: hypotension, moon face, buffalo hump, multiple purple striae on the flanks, proximal myopathy and oral candidiasis. He was admitted to our hospital with an initial diagnosis of hypercortisolism. Biochemical test results confirmed the diagnosis and revealed that he had elevated serum cortisol (8 a.m.) and ACTH levels on multiple samplings. Dexamethasone suppression test results were positive on two consecutive samplings. His urine cortisol level was elevated, but his vanillylmandelic acid and metanephrine levels were normal. Other laboratory tests were noncontributory to the diagnosis.

An MRI scan of his brain found no pituitary defects, but a spiral abdominal CT scan revealed bilateral adrenal hyperplasia. The clinical and imaging findings raised suspicion of an ACTH-producing tumor. A bronchoscopy and alveolar lavage was performed to investigate the patient's lungs, but no bronchial lesion was found.

**Figure 1:**
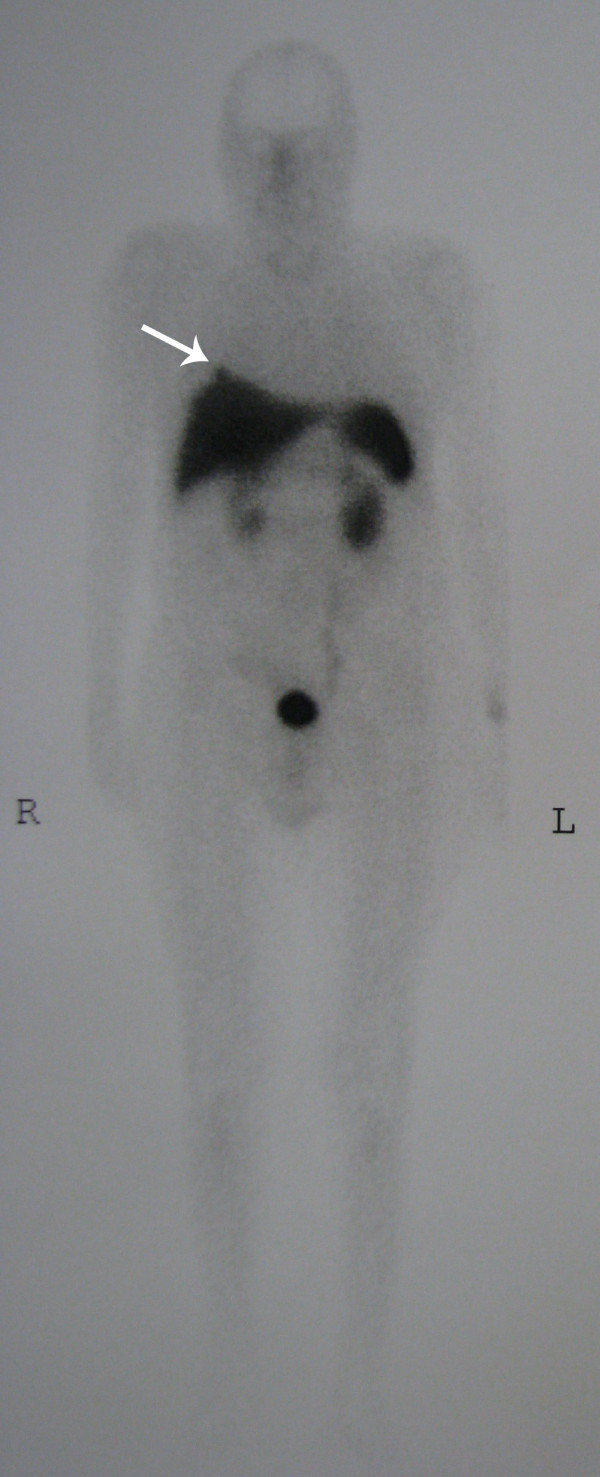
**Technetium-99m-labeled octreotide acetate scintigraphy in the whole body planar view**. This was performed 3 hours after injection of 15mCi technetium-99m-labeled octreotide acetate. There is a focal uptake in the lower lobe of our patient's right lung, just above his diaphragm, highly suggestive of an adrenocorticotropic hormone (ACTH)-producing bronchial tumor.

**Figure 2:**
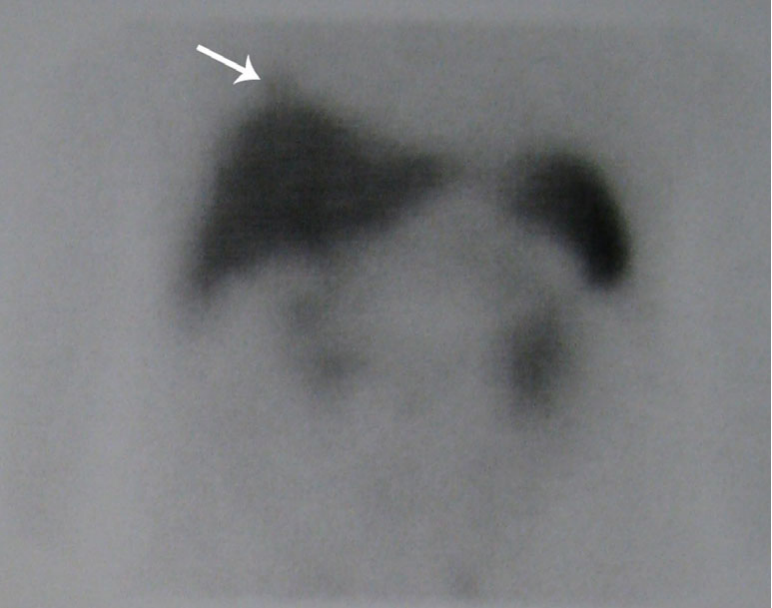
**Technetium-99m-labeled octreotide acetate scintigraphy in spot abdominal view**. This was performed 3 hours after injection of 15mCi technetium-99m-labeled octreotide acetate. There is a focal uptake in the lower lobe of our patient's right lung, just above his diaphragm, highly suggestive of an adrenocorticotropic hormone (ACTH)-producing bronchial tumor.

**Figure 3:**
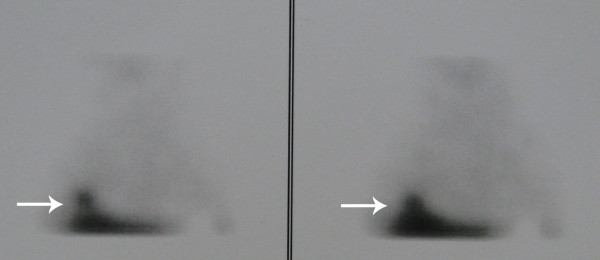
**Technetium-99m-labeled octreotide acetate scintigraphy performed in single photon emission computed tomography mode**. This was conducted 3 hours after injection of 15mCi technetium-99m-labeled octreotide acetate. The scan demonstrated a focal uptake in the lower lobe of our patient's right lung, just above his diaphragm, highly suggestive of an adrenocorticotropic hormone (ACTH)-producing bronchial tumor.

Technetium-99m-labeled octreotide acetate scintigraphy was performed in the whole body planar (Figures 1 and 2) and single photon emission CT mode (Figure 3), 3 hours after the injection of 555MBq (15mCi) technetium-99m-labeled octreotide acetate. The scan demonstrated a focal uptake in the lower lobe of the patient's right lung, just above his diaphragm, which was highly suggestive of an ACTH-producing bronchial tumor.

**Figure 4:**
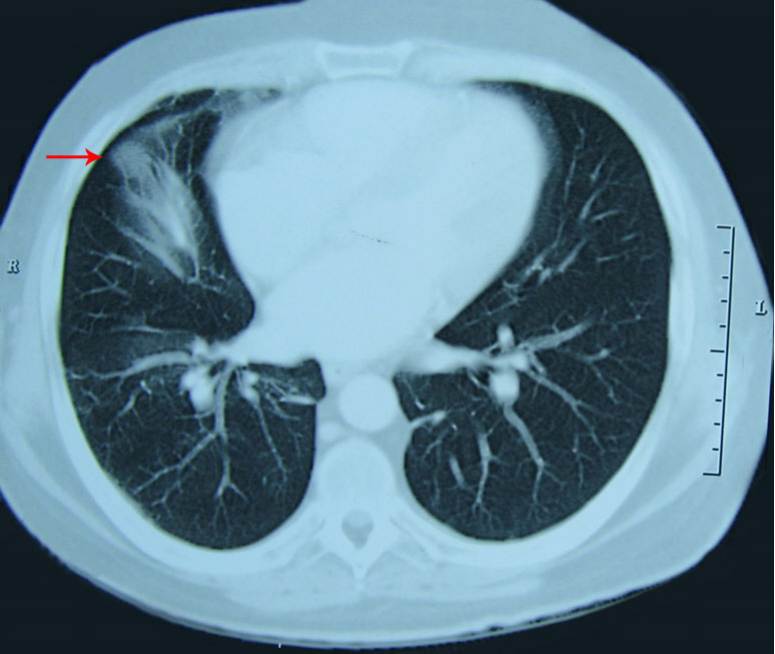
**Corresponding transverse images of a chest computed tomography scan showing a well-defined mass about 22 mm in the lower lobe of the patient's right lung**.

Corresponding transverse images from a chest CT scan showed a well-defined mass about 22 mm in diameter in the lower lobe of the patient's right lung (Figure 4).

A transthoracic biopsy was performed and histopathological evaluation established the diagnosis of a carcinoid tumor of the ectopic ACTH-producing type. After removal of the mass, the patient's condition improved significantly. His clinical symptoms diminished and the results of biochemical tests returned to normal ranges.

## Discussion

Ectopic ACTH-producing tumors occur in approximately 10% of cases of patients with Cushing's syndrome. Although a biochemical diagnosis of Cushing's syndrome is easily achieved, localization of the tumor is more difficult [1,3]. Cushing's disease is the cause of Cushing's syndrome in 70% of cases. A bronchial carcinoid tumor is the type of ectopic ACTH-producing lesion responsible in most cases [4,5]. Carcinoid tumors are malignant neoplasms originating from neuroendocrine cells [6]. To investigate the exact location of such tumors multiple imaging modalities are required, and at present no single modality can pinpoint the location of a suspected lesion [5,7]. The diagnostic utility of In-111 technetium-99m-labeled octreotide acetate scintigraphy in patients with suspected lesions has been debated. Some believe that it is not helpful [5], whereas others have reported radionuclide imaging to be a useful diagnostic tool [3].

From a literature review, we found limited studies have addressed the use of an octreotide compound with technetium labeling. Although In-111-labeled octreotide scintigraphy has been shown to be a helpful tool for the diagnosis of somatostatin-expressing tumors, and this method has been broadly used, it has several shortcomings such as high radiation dose, high cost and limited availability. To address these drawbacks, octreotide compounds have been labeled with Tc-99m.

In one study, the diagnostic outcomes of In-111 octreotide scintigraphy and Tc-99m Hynic Toc/Tate scintigraphy in 24 patients with different pathologies including two cases of ectopic Cushing's disease, were found to be identical [8].

In another investigation, the clinical value of tomographic technetium-99m-labeled octreotide acetate scintigraphy was compared with ^18^F-FDG dual-head coincidence imaging (DHC) of 44 patients with suspected lung tumors [9]. The sensitivity, specificity, positive predictive value, and negative predictive value of technetium-99m-labeled octreotide acetate scintigraphy were 100%, 75.7%, 90.1%, and 100%, respectively; and for ^18^F-FDG DHC the values were 100%, 46.1%, 83.8%, and 100%, respectively [9]. This comparison demonstrated that tomographic technetium-99m-labeled octreotide acetate scintigraphy had high sensitivity for distant metastases but lower sensitivity for the detection of hilar and mediastinal lymph node metastasis as compared with ^18^F-FDG DHC coincidence PET [9].

Our case report shows the usefulness of technetium-99m-labeled octreotide acetate scintigraphy in the localization of ectopic ACTH-secreting tumors in patients biochemically and clinically diagnosed with Cushing's syndrome. However, further well-designed studies to evaluate its efficacy are required.

## Conclusions

Our case report shows that technetium-99m-labeled octreotide acetate scintigraphy can effectively detect an ectopic ACTH-producing bronchial carcinoid.

## Competing interests

The authors declare that they have no competing interests.

## Consent

Written informed consent was obtained from the patient for publication of this case report and any accompanying images. A copy of the written consent is available for review by the Editor-in-Chief of this journal.

## Authors' contributions

AFE participated in the design and coordination of the study, drafting the manuscript and interpreting the radiological figures. MC participated in the design and coordination of the study, drafting the manuscript and interpreting the radiological figures. MHN participated in the design and coordination of the study, drafting the manuscript and interpreting the radiological figures. MS supervised the acquisition and interpretation of the radiological images. ME supervised the acquisition and interpretation of the radiological images. DB supervised the acquisition and interpretation of the radiological images. BF supervised the acquisition and interpretation of the radiological images. MA revised the article for important intellectual content and helped draft the manuscript. All authors read and approved the final manuscript.
